# Temporal Variation in Genetic Composition of Migratory *Helicoverpa Zea* in Peripheral Populations

**DOI:** 10.3390/insects11080463

**Published:** 2020-07-23

**Authors:** Omaththage P. Perera, Howard W. Fescemyer, Shelby J. Fleischer, Craig A. Abel

**Affiliations:** 1 Southern Insect Management Research Unit, USDA-ARS, Stoneville, MS 38776, USA; 2 Department of Biology, The Pennsylvania State University, University Park, PA 16802, USA; hif1@psu.edu; 3 Department of Entomology, The Pennsylvania State University, University Park, PA 16802, USA; sjf4@psu.edu; 4 Corn Insects and Crop Genetics Research Unit, USDA-ARS, Ames, IA 50011, USA; craig.abel@ars.usda.gov

**Keywords:** *Helicoverpa*, corn earworm, bollworm, SNP, population genetics, bollworm, genetic drift, DAPC

## Abstract

Migrant populations of *Helicoverpa zea* (Boddie) captured during 2002, 2005, 2016, and 2018 from Landisville and Rock Springs in Pennsylvania, USA were genotyped using 85 single nucleotide polymorphism (SNP) markers. Samples (n = 702) genotyped were divided into 16 putative populations based on collection time and site. Fixation indices (*F*-statistics), analysis of molecular variance, and discriminant analysis of principal components were used to examine within and among population genetic variation. The observed and expected heterozygosity in putative populations ranged from 0.317–0.418 and 0.320–0.359, respectively. Broad range of *F*_ST_ (0.0–0.2742) and *F*_IS_ (0.0–0.2330) values indicated different genotype frequencies between and within the populations, respectively. High genetic diversity within and low genetic differentiation between populations was found in 2002 and 2005. Interestingly, high genetic differentiation between populations from two collection sites observed in 2018 populations was not evident in within-site comparisons of putative populations collected on different dates during the season. The shift of *H. zea* population genetic makeup in 2018 may be influenced by multiple biotic and abiotic factors including tropical storms. Continued assessment of these peripheral populations of *H. zea* will be needed to assess the impacts of genetic changes on pest control and resistance management tactics.

## 1. Introduction

Insect species living in temperate climatic zones with broad geographic distributions and long-range migration capacity disperse seasonally throughout habitable geographic regions when favorable conditions arise. Migration allows species to exploit food sources, avoid competition, and find mates that immigrated from other populations to reduce inbreeding [1]. Admixtures of genetically diverse source populations from different regions to seasonal peripheral populations provide opportunities for robust exchange of genetic material that may be carried back to the source populations during reverse migration at the end of each warm season. The extent of this genetic admixture is dependent on multiple factors including the effective population sizes, temporal reproductive overlap, dispersal rate, growth rate, and food source preference [2,3,4]. This fundamental process influences genetic diversity and high gene flow rates between immigrants within peripheral populations from geographically separated source populations, leading to panmixis [3,5]. Genetic analyses of such species may reveal high genetic diversity and low genetic differentiation among source populations. Increased genetic diversity facilitates adaptations to changing environmental conditions and could lead to changes in biology and ecology including range expansion and establishment of permanent peripheral populations [6,7,8,9]. In contrast, widely distributed species with restricted gene flow rates may demonstrate high genetic differentiation and population sub-structure.

The polyphagous moth *Helicoverpa zea* (Boddie) (Lepidoptera: Noctuidae), commonly known as bollworm, corn earworm, or tomato fruitworm, is one of the several noctuid moths in the “heliothine complex” of pests in the Americas. *Helicoverpa zea* is a major pest of more than 300 plants species including economically important crop species such corn, cotton, soybean, and tomatoes [10,11,12]. Economic damage due to *H. zea* vary depending on the crop and the estimates are mostly based on an average of cost of control and crop losses of heliothine pest species. In 2019, approximately 1.3 million hectares of cotton cultivations (about 50% of total crop) was infested by heliothine pests and the total economic damage (loss plus cost of control) was estimated to be over $116 million [13]. The cost of control and yield loss due to damage averaged over the total cotton area grown (i.e., both infested and non-infested) in the United States in 2019 was $22.19 per hectare (ha) [13]. Soybean crop value lost due to *H. zea* in southern United States in 2012 was approximately $49 per ha, but could be greater with higher pest pressure [14,15,16]. In maize, *H. zea* causes damage to both field and sweet corn ears [17,18,19]. Impacts of ear damage in sweet corn is much higher due to low damage tolerance in fresh market sweet corn compared to field corn [20,21]. Combined damage and control costs for *H. zea* on cotton, corn, and soybean alone could exceed $100 per ha. Therefore, *H. zea* can be considered one of the most important pests of agriculture in North America.

Long range migration of *H. zea* within North America is well documented [22,23,24] and lack of synchrony between trap catches and emergence of overwintering moths have been used as evidence for seasonal migration of this species from Mexico and south Texas to northern latitudes of the United States [25,26,27]. The ability of *H. zea* to disperse over long, medium, and short ranges provides this species with several advantages including avoiding competition for food resources by following crop planting schedules during the growing season, reproducing with genetically diverse mates to maintain population genetic diversity, and adaptations to changing environmental and climatic conditions. With widespread planting of transgenic crops expressing *Bacillus thuringiensis* toxins (Bt crops) across the continent [28], *H. zea* is constantly under pressure to overcome deleterious effects of these Bt proteins. With high genetic diversity and panmixis in southern US populations [29,30], this species has the potential to adapt to various Bt toxins and rapidly spread resistance alleles across populations. In fact, reports on control failures in Bt crops and potential field evolved resistance indicate that *H. zea* could be rapidly evolving resistance under the intense selection pressure from widespread use of Bt toxins in its host plants [31,32,33,34,35,36]. Coordinated efficacy trials with Bt-sweet corn has documented field-evolved resistance to several Cry proteins, and the utility of studies conducted in peripheral populations to detect these changes [37]. Although information on dispersal, genetic diversity, and gene flow of *H. zea* populations is necessary to evaluate the potential to spread genetic changes that may contribute to tolerance to Bt toxins across the distribution of this species, there is a paucity of data and resources available to conduct population genetic studies on this important pest species. Our laboratory previously developed microsatellite markers for this species using resources available at the time [38] and conducted population genetic studies on source populations from the southern USA [30], and sink populations from Landisville (LV), and Rock Springs (RS), PA in the northeastern USA [39]. The populations from LV and RS, collected in 2002 and 2005, were primarily made of moths dispersed from other source populations or recent progeny of those migrants breeding in sink populations. Sample collection data and microsatellite analysis in Seymour et al. [39] indicated distinct moth emergence or flight patterns and high genetic diversity in both sites, respectively, but no population genetic structure was detected within or among populations collected from two sites. High genetic diversity observed in this study was attributed to the presence of migrants (or their progeny) from different source populations having different allele frequencies. Absence of genetic structure in LV and RS populations indicated high rates of gene flow and panmixis in *H. zea* populations contributing migrants to these sink populations. The analysis of peripheral (sink) populations suggested that centers of source populations contributed many founders to the sink populations [40]. In general, source populations tend to have higher abundance and fitness than sink populations. Therefore, evolution by natural selection is expected to be stronger for maintaining or improving adaptations in source populations [41] and study of a few peripheral sink populations will capture the changes to genetic makeup in source populations.

With recent reports of control failures on Bt crops in the southern and eastern USA, evaluation of *H. zea* populations was necessary to identify potential changes to genetic structure of this species. Our goals in this study were to (1) develop a validated set of single nucleotide polymorphism (SNP) markers that will provide higher resolution population genetic parameter estimates than microsatellite markers and (2) compare genetic makeup of recently collected populations from LV and RS with those collected in 2002 and 2005 from the same sites to evaluate changes to genetic makeup of *H. zea* over time. This study uses a panel of SNP markers to evaluate genetic composition changes over time and space in *H. zea* from two peripheral populations collected 11 to 16 years apart. We postulate that comparison of samples collected from sink populations before and after widespread control failures in Bt crops could detect genetic shifts in source populations. In addition, this study will establish baseline sink genetic structure data for *H. zea* and the SNP markers validated here will facilitate future population genetic studies of this species.

## 2. Materials and Methods 

### 2.1. Sampling

**Figure 1:**
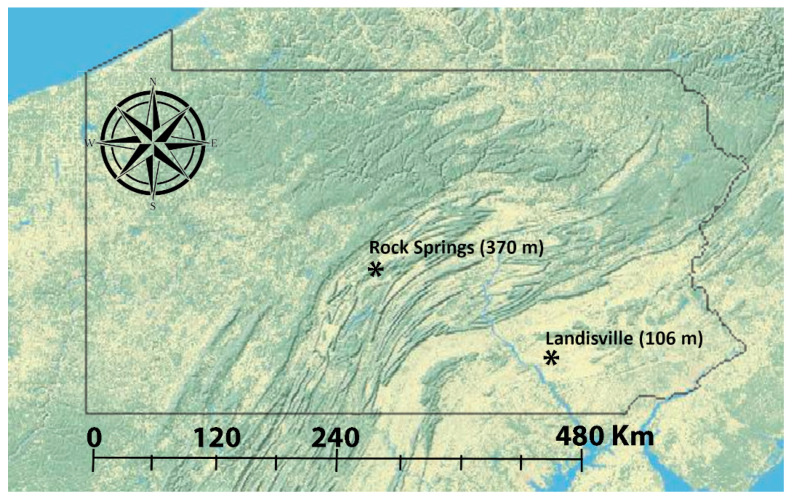
A topographic map with state of Pennsylvania outlined and the collection sites in Landisville (LV) and Rock Springs (RS), marked. Linear distance between collection sites is approximately 150 km. Elevation of Landisville and Rock Springs are 106 m and 370 m above mean sea level, respectively. Topological map was obtained from the US Geological Survey (https://ngmdb.usgs.gov/topoview/viewer/; accessed on 05 June 2020).

Adult male *H. zea* were sampled near corn fields using Hartstack pheromone traps [42] from August through September during 2002, 2005, 2016, and 2018 near the Russel E. Larson Research and Education Center at Rock Springs (RS), Pennsylvania, USA (40°42′38.1″ N 77°57′52.2″ W) and the Southeast Research and Extension Center at Landisville (LV), Pennsylvania, USA (40°07′04.7″ N 76°25′30.5″ W) (Figure 1). Linear distance between the collection sites is approximately 150 km, separated by a series of mountain ridges with elevation up to 620 m above mean sea level (MSL). Rock Springs and Landisville are approximately 365 and 120 m above MSL, respectively. Moths removed from traps were stored at −20 °C. Genomic DNA was extracted from the thorax and head of insects using MasterPure DNA extraction reagents (Epicentre, Madison, WI, USA). Sample sizes and dates of insect collections from LV and RS in 2002, 2005, 2016, and 2018 are given in Table 1.

### 2.2. Single Nucleotide Polymorphism Marker Development

Genomic DNA samples extracted from 95 F_1_ progeny insects from a cross between a laboratory colony maintained at US Department of Agriculture (USDA) Southern Insect Management Research Unit, Stoneville, MS and an insect collected on a wild host in Stoneville, MS were submitted to a Cornell University Genotype-by-Sequencing (GBS) service facility to identify SNPs using the protocol described in Elshire et al. [43]. Briefly, genomic DNA double digested with restriction enzymes EcoR I and Pst I was used to construct the GBS sequencing libraries. The The GBS library containing a fraction of the *H. zea* genome was sequenced on single lane of a flowcell on Illumina HiSeq 2000 instrument to obtain 100 nucleotide single end reads. Tassel (v3.0.147) [44] was used to call SNPs from the sequenced GBS library with options listed in Supplementary data Table S1. VCFtools (v0.1.10) [45] was used to filter and summarize data, and to generate input files for multidimensional scaling (MDS) using PLINK (v 1.9) [46]. Scaffolds from a genome assembly of *H. zea* [47] was used as the reference for alignment and to identify SNP containing sequence tags. SNPs were verified by mapping a portion of the original Illumina sequence reads used to generate the genome assembly [47] and 384 computationally verified SNPs were manually selected to cover 384 longest scaffolds. Primers developed for SNP containing sequences were used to verify the ability to amplify loci from genomic DNA isolated from a laboratory colony insect and 96 validated SNPs were selected to synthesize reagents for SNP genotyping on a Fluidigm EP1 genotyping platform (Fluidigm Corporation, San Francisco, CA, USA).

### 2.3. Single Nucleotide Polymorphism (SNP) Analysis

DNA extracts from field collected samples were quantified using a Qbit Flex fluorometer and Quanti-iT PicoGreen assay kit (Thermofisher Scientific, Waltham, MA, USA) for double stranded DNA and adjusted to 60 ng/µl prior to SNP genotyping with the Fluidigm EP1 system using integrated fluidic circuits (IFCs) that can analyze 96 samples with 96 SNP markers. DNA from one laboratory control and 95 insects from each collection was genotyped using the Fluidigm EP1 instrument. Genotype data files were imported to Fluidigm Genotyping Analysis v4.1.3 software and genotypes were exported as comma separated text files that were opened in Microsoft Excel 365 for further processing. Nucleotide symbols in genotype data were converted to the two-digit numerical format of 01, 02, 03, and 04 to represent A, C, G. and T, respectively, to produce input data for analysis programs that use numerical input.

### 2.4. Genetic Structure

The number of insects collected in pheromone traps in LV and RS vary depending on flight times of local populations and influx of dispersing insects. Therefore, initial analysis was performed by dividing the samples into 16 partitions (putative populations) based on the collection year and time of the collection (Table 1). Subsequent analyses were performed by pooling samples by collection year and cluster assignments resulting from further analyses (see below). Genetic and statistical analyses were performed using the programs ARLEQUIN 3.5 with R functions [48] and R version 3.6.1 (R Core Team 2015). Samples with 10% or more missing loci were removed from the analysis. ARLEQUIN 3.5 was used for estimating most population parameters such as expected (*H*_E_) and observed (*H*_O_) heterozygosity, within (*F*_IS_) and among (*F*_ST_) population fixation indices, pairwise *F*_ST_, and for analysis of molecular variance (AMOVA). Deviations from Hardy–Weinberg equilibrium (HWE) across all populations using an exact test [49], based on Markov Chain iterations with 100,000 steps and 10,000 dememorization steps was implemented in ARLEQUIN 3.5.

STRUCTURE 2.3.4 [50] was used to perform a Bayesian clustering analysis using a burn-in of 50,000 iterations and 500,000 Markov Chain Monte Carlo (MCMC) simulation for K (populations or clusters) from 1 to 10 under the admixture model with prior information on population collection sites provided. Ten simulations were performed for each K value and the resulting data were analyzed using the Evanno method [51] implemented in the Structure Harvester online tool (http://taylor0.biology.ucla.edu/structureHarvester/; accessed on 07 May 2020) [52] to identify potential genetic clusters. The rate of change in the log probability of data between successive K values is calculated by the Evanno method to obtain the ad hoc statistic ΔK. The K value with the highest ΔK represents the number of potential genetic clusters in the population. After selecting the best K-value using the initial ΔK, the STRUCTURE program was run using 20 replicates for each K value from 1 to 8 with 100,000 burn-in replicates followed by 1,000,000 MCMC replications to recalculate ΔK.

Discriminant analysis of principal components (DAPC) [53] implemented in the R package adegenet [54] was used to determine population genetic structure of *H. zea* using prior population assignments as well as no prior assignments. The DAPC analysis with non-prior population assignment was carried out to evaluate number of putative clusters (K) between two and 40. The Bayesian information criterion (BIC) was then used to evaluate the relevance of different K values to population structure. Assignment values for the selected number of clusters were then generated for each individual using DAPC [53]. DAPC first transforms the data using principal components analysis, which ensures that the variables are not correlated and that the number of variables is smaller than the number of individuals. Then, discriminant analysis partitions the variance into among and within group components, maximizing separation between groups. DAPC does not assume a population genetics model and it is not constrained by Hardy–Weinberg or linkage equilibrium assumptions, making it a robust method to test for genetic differentiation of populations. Sixteen putative *H. zea* partitions corresponding to the date and location of collection were assigned to the prior population set. All statistical analysis results are reported as mean ± standard deviation (s.d.).

## 3. Results

### 3.1. SNP Discovery

Sequencing of the GBS partial genomic library yielded a total of 252,303,111 reads that had 178,205,561 good barcoded reads. Filtering resulted in 894,576 tags, of which 601,201 (67.2%) and 58,752 (6.6%) were aligned to unique and multiple positions of the *H. zea* genome, respectively, and 234,623 reads (26.2%) could not be aligned. Since we used adult insects to obtain genomic DNA for the GBS library, microbial contaminants present in the tissue pieces used (e.g., gut and exoskeleton) may have contributed to the sequence tags that could not be mapped to the genome. Mean coverage depth per individual was 77.8 ± 9.9 and the depth per site was 61.9 ± 64.2. Mean missingness for individuals and sites were 0.28 ± 0.10 and 0.28 ± 0.30, respectively. Genotypes with sequencing depth between 3 and 127 were selected for further analysis and 6,891 biallelic SNPs with more than 20% missing data were filtered out. After Tassel pipeline analysis, a total of 15,669, 7477 and 2664 VCF, unfiltered hapmap, and filtered hapmap SNPs, respectively were detected within GBS reads from 95 *H. zea*. Compared to other species of insects, the low number of SNPs most likely resulted from high repetitive DNA content from the EcoR I/Pst I double digest that was excluded from GBS library construction and low levels of genetic polymorphism in the parents used in generating F1 individuals used in the study. In addition, filtering for loci with multiple alleles and missing data may have further reduced the number of useable SNPs identified in this study. Nevertheless, the number of SNPs recovered was sufficient to develop assays for population genetic studies. A total of 384 polymorphic SNPs were manually selected to assign one SNP per scaffold in the largest scaffolds that had a computationally validated SNP for developing assays. Primers developed for the SNP loci were used to verify amplification of *H. zea* genomic DNA using real time polymerase chain reaction (PCR). After PCR verification, 96 loci were selected to develop Fluidigm SNP assays. The SNPs were developed for the unpublished version 6 of the *H. zea* genome, assembly version 5 was published [47]. Therefore, scaffolds of genome assembly 6 annotated with SNPs were submitted to GenBank under accessions MT702886 to MT702981. Locus names, flanking nucleotide sequences of SNPs, type of SNP and the corresponding scaffold numbers of these 96 SNP loci in the genome assembly versions 5 (PRJNA37843) and 6 are also provided in the Supplementary Table S2.

### 3.2. SNP Analysis

After a preliminary analysis of data using ARLEQUIN, loci and samples with more than 10% missing data in one or more populations and non-informative monomorphic loci across all populations were excluded from further analysis to retain a total of 85 SNP loci. Initial data set and the final genotype data used in this analysis are given in Tables S3 and S4, respectively. The number of samples in partitions ranged from 20 to 114 with a total of 351 samples each from two collection sites (Table 2).

Observed and expected heterozygosities were calculated by pooling all 702 and samples ranged from 0.0014 (locus Hz6-105) to 0.871 (Hz6-406) and 0.0014 (Hz6-105) to 0.500 (Hz6-954), respectively. Mean observed heterozygosity in putative populations ranged from 0.3168 ± 0.2334) in 05LV-Aug24 to 0.4181 ± 0.2586 in 18LV-Aug06 and mean expected heterozygosity ranged from 0.3203 ± 0.1570 in 05LV-Aug19 to 0.3596 ± 0.1636 in 18LV-Aug06 (Table S5a). Relatively high standard deviation values indicate high variation in expected and observed heterozygosity estimates in all putative populations. Locus-by-locus observed and expected heterozygosity for 16 partitions are given in Table S5b. Total number of transitions, transversions, substitutions, private substitution sites, and the molecular diversity for the 16 partitions and the mean and standard deviation for all partitions are given in Table S6. The number of transitions ranged from 42 to 53 with a mean value of 46.7 ± 3.1 and the number of transversions ranged from 19 to 23 with a mean of 21.2 ± 1.4. The total number of substitutions ranged from 62 to 77 (mean 67.9 ± 4.1). Molecular diversity indices (Pi) for individual populations ranged from 24.1 to 29.9 with a mean of 25.8 ± 1.7.

Exact tests conducted across individual loci in pooled populations indicated significant deviations from HWE in 39 loci (*p* ≤ 0.01). However, deviations from HWE at these loci were inconsistent in exact tests performed on individual populations (Table S7). Individual removal of each of these loci from the analysis did not significantly influence the genetic structure of the *H. zea* populations. Exact tests based on genotype frequencies for differentiation [49,55] indicated that there are no significant differences among putative populations (*p* ≤ 0.01; exact *p* value for non-differentiation (0.8000 ± 0.1472; Table S8). The inbreeding coefficient, *F*_IS_, in 16 putative populations ranged from 0.0 to 0.2330, but only the coefficients for populations 05LV-Aug24 (0.1851) and 05LV-Sep03 (0.1467) significantly deviated from zero at *p* ≤ 0.05 (Table 2), indicating random mating across all putative populations. The observed *F*_IS_ may have been influenced by immigrants that originated from populations with different allele frequencies captured in pheromone traps.

**Figure 2:**
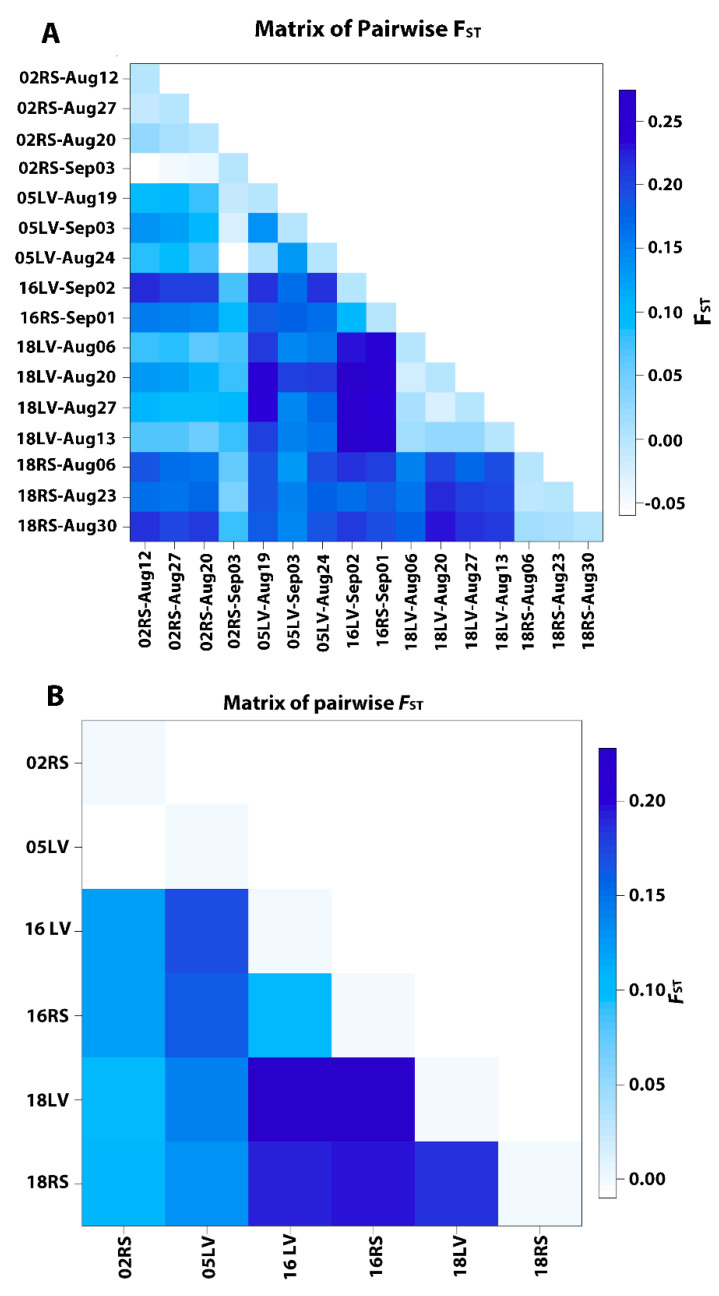
Matrix of pairwise *F*_ST_ estimates for *Helicoverpa zea* collected from Landisville (LV) and Rock Springs (RS), PA, partitioned into 16 putative populations by collection date (**A**) and pooled into six populations by collection year (**B**). Putative population names use the convention of two-digit collection year (02, 05, 16 or 18), two letter collection site code, followed by collection month and date. Intensity of blue color squares corresponds to the *F*_ST_ values shown in the scale bar. Corresponding data are provided in Supplementary Data Table S9a,b.

**Figure 3:**
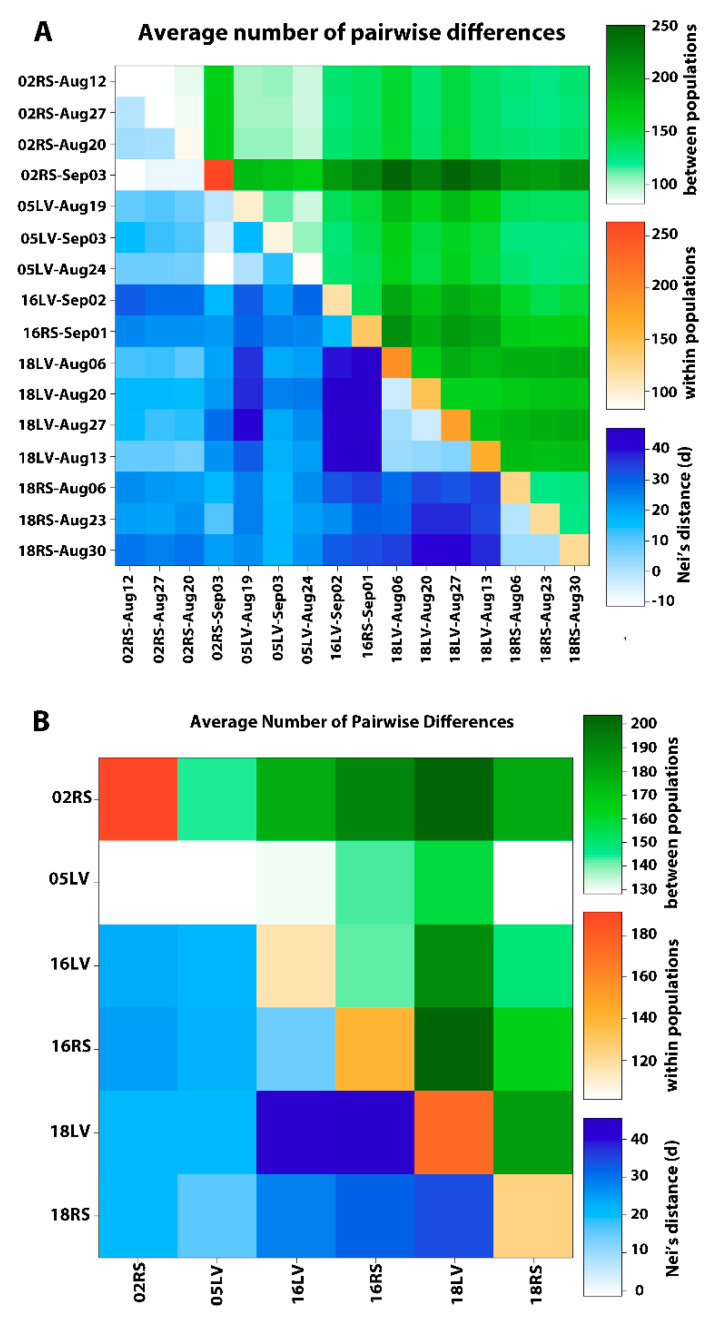
Average pairwise differences between populations (above diagonal), within each population (diagonal) and Nei’s genetic distances (55) between populations (below diagonal) of *H. zea* samples partitioned by collection date within each year (**A**) and pooled by collection year and collection site (**B**) for populations from Landisville (LV) and Rock Springs (RS), PA. Putative population names use the convention of two-digit collection year (02, 05, 16 or 18) and two letter collection site code. Samples partitioned by collection date within year are also indicated by three-letter month code and the date. Increasing intensity of colors indicate increasing number of differences. Corresponding data are provided in Supplementary Data Table S10a,b.

Pairwise *F*_ST_ between putative population pairs collection data ranged from 0.0 to 0.2742 (Figure 2A and Table S9a) and the global *F*_ST_ was 0.01 with an *F*_IS_ of 0.16. Pairwise genetic distance comparisons (Table S10a,b) indicated that RS populations collected in 2002 had the least differences among collection dates with non-significant *F*_ST_ values (Table S9a). Average pairwise differences between populations and within populations as well as Nei’s genetic distances [56] between populations of *H. zea* are shown in Figure 3. Populations partitioned by collection date indicate that within population differences (diagonal) were highest in the 02RS-Sep03 population (263.4) followed by LV populations collected in 2018 having values ranging from 140.2 to 194.9. Remaining populations had within population genetic differences from 82.5 to 126.1 (Figure 3A and Table S10a). When data for each site was combined by collection year, 02RS population had the highest number of within population differences (191.5) followed by 18LV and 16RS with 174.0 and 140.2, respectively (Figure 3B and Table S10b). Pairwise differences and Nei’s genetic distance between populations reflected the pattern of *F*_ST_ estimates for the population pairs.

RS populations collected on 12 and 20 August 2002 and LV populations collected on 19 and 24 August 2005 had relatively low *F*_ST_ estimates ranging from 0.0734 to 0.0908 (Table S9a). The population pair 02RS-Sep03 and 05LV-Sep03 did not show any differentiation. However, *F*_ST_ estimates for 02RS populations paired with 16RS and 18RS populations significantly deviated from zero (0.1486 to 0.2117). Comparison of LV population pairs from 2005 resulted in non-significant *F*_ST_ for the pair 05LV-Aug19 and 05LV-Aug24, but significantly deviated from zero (*p* ≤ 0.01) when compared with 05LV-Sep03. *F*_ST_ estimates for 05LV populations paired with LV populations from 2016 and 2018 (16LV- and 18LV-) were significantly high ranging from 0.1325 to 0.2365 (Table S9a). Interestingly, all pairwise *F*_ST_ estimates among LV or RS populations collected in the month of August 2018 were essentially zero, indicating non-differentiation. However, all comparisons between LV and RS populations collected in 2018 were significantly high (0.1529 to 0.2319; *p* ≤ 0.01). In contrast, *F*_ST_ estimate for 02RS-Sep03 and 05LV-Sep03 was non-significant and that for 16LV-Sep02 and 16RS-Sep01 was 0.0996. When analysis was performed by pooling all samples collected within a year from each site into 6 populations, *F*_ST_ for 02RS and 05LV pair was essentially zero, but *F*_ST_ values for all other pairs ranged from 0.0996 between 2016 LV and RS populations to 0.2280 between LV populations collected in 2016 and 2018 (Figure 2B and Table S9b). *F*_ST_ values for 02RS and 05LV populations paired with LV and RS populations from 2016 and 2018 ranged from 0.1003 to 0.1710. The *F*_ST_ for 2018 populations from LV and RS was significantly high (0.1852) compared to the estimate for the LV and RS populations from 2016 (Table S9b).

Linkage disequilibrium (LD) was detected between several pairs of the SNP loci used in the final genetic analyses, but the number of linked loci varied across 16 putative populations (Table S11 and Figure S1). No linkage was detected for loci Hz6-105, Hz6-148, Hz6-875, and Hz6-1790 in any one of the putative populations. *Helicoverpa* species have 31 haploid chromosomes [57] and physical linkage is expected between most of the 85 loci used in this analysis. Although analyses based on human and *Drosophila melanogaster* Meigen (Diptera: Drosophilidae) genomes indicate that LD due to physical proximity may not exist beyond a few thousand base pairs [58], haplotype blocks bound by recombination hotspots may extend over 100 Kbp [59,60,61]. All or most SNPs within these haplotype blocks may show high LD [62,63,64,65,66]. Although low density of 85 SNPs across the approximately 350 Mbp genome of *H. zea* used in this study is not sufficient to identify haplotype blocks or genomewide associations (GWAS), LD observed here may indicate unrecognized sub-structuring in populations analyzed in this study.

### 3.3. Genetic Structure

**Figure 4:**
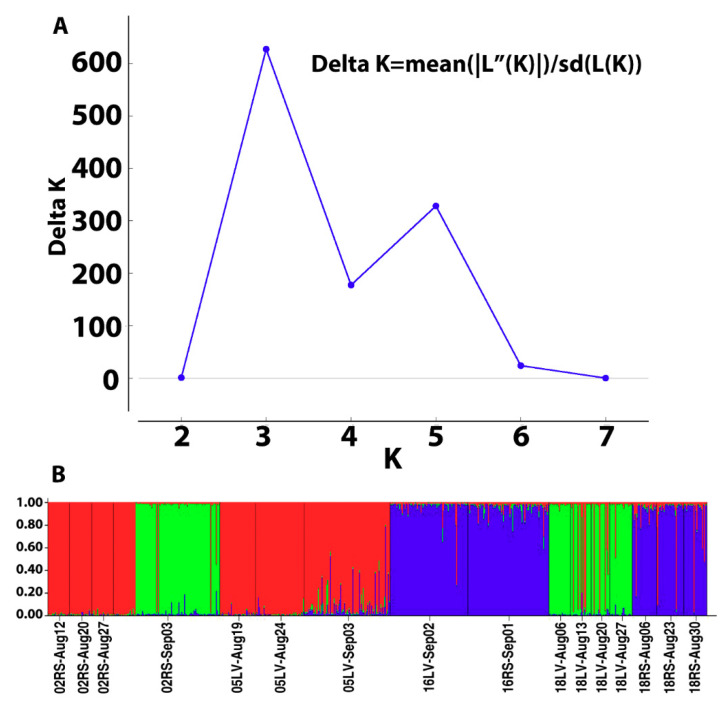
The plot of ΔK for successive population cluster number (K) generated using the Evanno method [50] implemented in the Structure Harvester web application (**A**), and the bar plot generated using population assignments in one of the STRUCTURE [49] simulations at K = 3 (**B**). Putative population names are marked on the X-axis.

**Figure 5:**
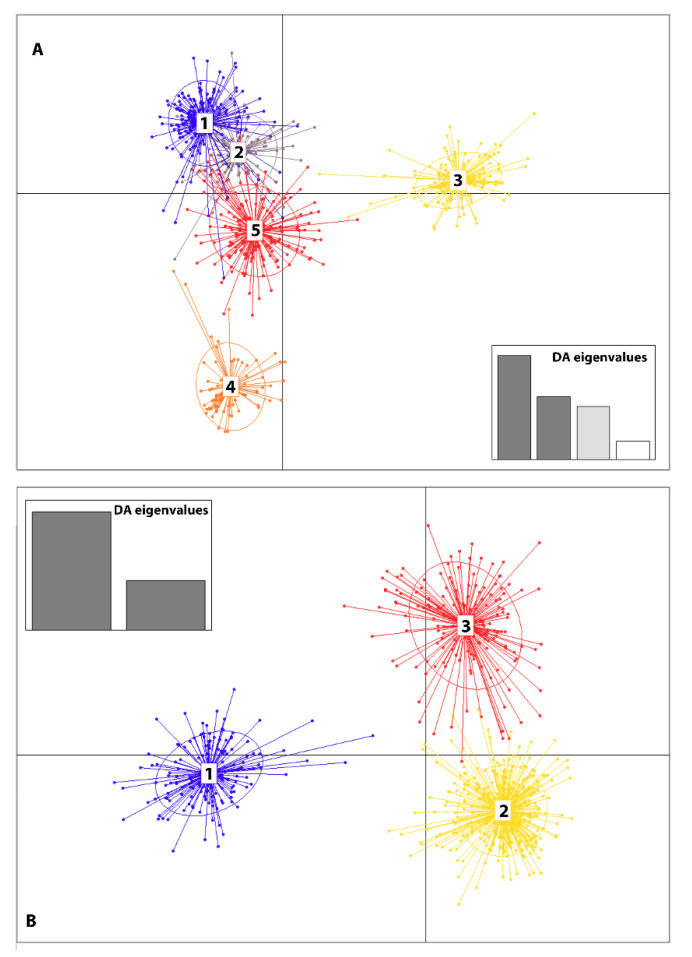
Most parsimonious cluster number estimation using discriminant analysis of principal components (DAPC) iterations from K = 1 to 40 to obtain the successive Bayesian information criterion (BIC) of K = 8 and the putative genetic clusters generated by DAPC using the estimated BIC value of eight (**A**). DAPC results using prior assignment of three putative populations based on STRUCTURE results (**B**). Both plots show the first two axes of the analysis (inset plot). Each color represents a unique population cluster with the corresponding circles showing the prior unique groupings of the 13 putative populations.

Simulations with STRUCTURE resulted in ΔK peak reaching a value 623 at K value of 3, indicating three population clusters (Figure 4A). Depicted as red, green and blue in the bar chart (Figure 4B). Non-prior population DAPC analysis based on differences between successive values of BIC summary statistics and successive cluster assignment from repeated DAPC runs from K = 1 to 40 [53,54] revealed K = 8 as the most parsimonious number of clusters (Figure S2). This additional clustering did not correspond to any significant structure within putative populations or collection time (Figure 5A). When DAPC was performed using a prior assignment of three putative populations corresponding to STRUCTURE analysis, three more distinct genetic clusters without any clear relationship to collection year or location emerged (Figure 5B).

Global tests of sample differentiation based on genotype frequencies [49,55,67] performed with 20,000 Markov steps generated a non-differentiation exact *p*-value of 0.4626 ± 0.1682 and tests for differentiation between all pairs of samples resulted in *p*-values greater than 0.2383, indicating no significant substructure in putative populations. Global AMOVA performed with ARLEQUIN as a weighted average over loci from the 16 putative populations indicated that high genetic diversity in individuals within the total population contributed to 96.89% of the total variation with an *F*_IT_ of 0.031 (*p* = 0.00001) and non-significant inbreeding coefficient (*F*_IS_) of −0.058 (essentially 0; *p* = 1.00). AMOVA performed by partitioning the genotypes into groups based on collection time, location or cluster assignments resulted from DAPC analysis also indicated that greatest molecular variance occurred in individuals within the total population (Table 3). AMOVA performed by partitioning the putative populations only based on the collection date and place (i.e., disregarding the collection year) indicated high genetic variability in collections made from August 30 to September 3 of each year compared to earlier collections. Regardless of the type of population partitioning, AMOVA indicated that genetic variation among individuals within populations was minimal and not significant while that of individuals was significant compared to total population.

## 4. Discussion

Previous population genetic studies of *H. zea* based on microsatellites conducted in our laboratory and other studies based on restriction fragment length polymorphism and biochemical markers have indicated low genetic differentiation in regional *H. zea* populations within the Americas [29,30,39,68,69,70,71], but high genetic diversity due to migration of insects from source populations with different allele frequencies to “sink” populations, which may or may not be peripheral, during the growing season [39]. Low *F*_ST_ and high *F*_IS_ values observed in Seymour et al. [39] suggested that the insects captured in pheromone traps originated from breeding populations with different allele frequencies. The observations in Seymour et al. [39] led to the postulation that migration of *H. zea* to peripheral populations is asymmetrical.

In the present study, we used 85 SNP loci to estimate genetic parameters of insects collected in 2002, 2005, 2016, and 2018 from two peripheral habitat sites in Pennsylvania. Non-significant *F*_ST_ estimates observed for all putative populations collected in RS during 2002 indicated a panmictic population of *H. zea* with high rates of gene flow. However, at LV in 2005, the influx of genetically diverse populations from overwintering core populations may have contributed to low-level genetic differentiation between putative populations collected in August and September (*F*_ST_ from 0.0036–0.1356). Little differentiation observed between putative populations collected in September of 2016 at both LV and RS indicated there was some gene flow between two sites. Insects collected at both sites during the 2018 growing season showed no genetic differentiation among putative populations collected on different dates within each site. When populations collected in different years within the same site were compared, *F*_ST_ values for 05LV/16LV, 05LV/18LV, and 16LV/18LV pairs were 0.1710, 0.1392, and 0.2280, respectively. Similarly, comparison of RS population between years yielded *F*_ST_ estimates of 0.1237, 0.1068, and 0.1950 for 02RS/16RS, 02RS/18RS, and 16RS/18RS pairs, respectively. Comparisons of populations from two sites collected within the same year yielded *F*_ST_ estimates of 0.0996 and 0.1852 for 16LV/16RS and 18LV/18RS population pairs, respectively. Overall, within a site, populations that paired more recent times (2016 versus 2018) yield higher FST estimates than pairs that used 02 or 05 years. Similarly, across sites, populations with the most recent times (2018) yield higher *F*_ST_ estimates. Relatively high *F*_ST_ estimates between populations from LV and RS in 2016 and 2018 and lower *F*_ST_ values for other comparisons outlined above may point to a change in genetic composition of *H. zea* populations in recent years. Analyses conducted by partitioning the populations by collection date indicates that there is a temporal shift in genetic composition within each site during each year, with insects collected in late August and September of 2016 and 2018 exhibiting higher differentiation than those from 2002 and 2005. It is possible that mountainous terrain may act as a physical barrier to restrict intraspecific gene flow that might result in significant genetic differentiation within and between years. However, determining the actual forces that influenced recent genetic differentiation between LV and RS populations could not be done without a detailed study of intrinsic and extrinsic factors, but one can only speculate many possible reasons including change in crop landscape and pest management practices as well as insect dispersal patterns influenced by different airflow patterns due to changing climatic conditions.

Long-range dispersal in heliothine species is well documented [26,72,73,74] and high gene flow in *Helicoverpa* species may contribute to low genetic differentiation among populations across large geographical regions [39,74,75,76,77,78]. However, reported temporal and spatial genetic differentiation, possibility due to limited gene flow, for closely related *Helicoverpa armigera* (Hübner) (Lepidoptera: Noctuidae) in Australia and India [79,80,81,82], indicates the possibly of this for *H. zea*. A previous microsatellite-based population genetics study of *H. zea* using insects collected in 2002 and 2005 from LV and RS concluded asynchronous influx of migrants from core populations, contributing to low genetic differentiation and high genetic diversity [39]. This deduction is in contrast to the studies on the same species from the southern USA (e.g., Alabama, Mississippi, Texas) that indicated low genetic differentiation as well as limited genetic diversity in southern source populations [29,30,68]. Although there is a paucity of genetic studies on insect populations overwintering in southern regions of North America that migrate during the warm season, it is possible that genetically diverse core populations from multiple geographical locations (e.g., southeast and eastern seaboard) may be the source of high genetic diversity in migrant populations. By analyzing seasonal migratory populations in sink populations, we may be capturing, in addition to genetic changes in source populations, the effects of changes in weather, wind patterns, and dispersal rates from different source populations. These variations could lead to high genetic diversity as well as high population differentiation reflected in *F*_ST_ estimates due to differences in genotype frequencies between populations.

The migration of another noctuid, the fall armyworm, *Spodoptera frugiperda* (J.E. Smith) (Lepidoptera: Noctuidae), has been modeled using coupled bio-physical processes, and validated with SNPs that function as markers of natal origins [83,84]. The overwintering range for fall armyworm is restricted to Texas and Florida. The models and SNPs document northerly migration, with the Texas-source populating the continental interior and extending to the Appalachian Mountains and northeastern US, and the Florida-source repopulating the Atlantic coast and southeastern areas. Migration patterns were influenced by nocturnal air-flow trajectories, with annual variation in the degree of mixing of Texas and Florida source populations [83,84,85]. *Helicoverpa zea* may be using similar nocturnal wind currents during migration, albeit from an overwintering range that is much wider and in closer proximity to our study sites. The high genetic differentiation between 2018 LV and RS populations compared to other years may reflect the variation in aerobiological processes [86] resulting in dispersal of insects from new genetically diverse source populations or stronger within season differentiation of migratory populations at each site during growing season in 2018. Also, the two sites are 150 km apart and separated by the Appalachian mountain range. Relatively low altitude (120 m above MSL) makes it easy for insects from the eastern seaboard to migrate to LV, and trap captures tend to occur earlier and are higher at the LV site than the RS site. In contrast, the high mountain range may serve as a physical barrier to insect movement between LV and RS, unless carried by high altitude wind currents. Lack of variation observed between LV and RS populations collected in 2005 and 2002, respectively, in this study and between 2005 populations from two sites studied in Seymour et al. [39] indicate that avenues for gene flow between LV and RS may have been present during that time. A review of weather data indicated that there were no major hurricanes or tropical storms that affected the eastern seaboard in 2002 and 2005. However, one tropical storm and one hurricane affected this seaboard from 30 May to 12 July 2018 (Figure S3; https://www.ncdc.noaa.gov/monitoring-content/sotc/tropical-cyclones/2018/annual/tws_atl_latest.gif; accessed on 6 July 2020). It is possible that high winds from these storms could have forced source populations with different genotype frequencies from coastal areas toward LV to alter the genetic composition of that population. However, it is not possible to accurately predict the causes of high *F*_ST_ estimates between LV and RS populations without developing prediction models that combine weather data with extensive genetic and ecological datasets.

In summary, this study generated a set of SNP markers suitable for population genetic studies and identified high genetic diversity and low genetic differentiation in *H. zea* populations collected at RS in 2002 and at LV in 2005, but populations collected in 2018 from LV and RS exhibited high genetic differentiation. The populations within each site had no genetic differentiation. DAPC and STRUCTURE analyses indicate some sub-structuring in *H. zea* populations, in contrast to previous studies that did not show population structure in *H. zea*. Although *H. zea* are capable of dispersal over a large geographic range which contribute to genetic homogenization and panmixia [30,39], genetic differentiation in populations between two sites, but not within sites in 2018 indicate that either the accumulation of genetic variance within year due to random genetic drift, adaptation to local environmental conditions or control practices or sources of genetic stock contributing to the populations may have changed, either temporarily or permanently, due to unknown factors. The temporal and spatial genetic differentiation observed in this study has not previously been observed for in *H. zea* populations and there may be a multitude of underlying factors that may have contributed to this change, including environmental, climatic, and genetic factors. Yearly variations in gene flow rates in *H. armigera* influenced by various factors have been reported [81,82] and *H. zea* populations may be undergoing such variations in geneflow. These changes are likely transient within a year, and by comparisons within 2002 and 2016, significance in local variation among these sites is not consistent. Inconsistency could be influenced by effective population sizes that in turn impact the effects of random genetic drift at each site and gene flow between LV and RS. If high genetic differentiation observed between populations collected in 2018 is not a temporary phenomenon, *H. zea* management practices, crop biotechnology, and insect resistance management models may be impacted.

## 5. Conclusions

Population genetic analyses of *H. zea* populations from two sites about 150 km apart in Pennsylvania, USA collected in 2002, 2005, 2016, and 2018 indicated a temporal variation in genetic composition of insects collected in 2016 and 2018. Population genetic parameter estimates suggested a trend toward population differentiation, although it may be a transient or temporary phenomenon. This shift may be a result of several factors including annual changes in migratory patterns, climatic conditions, wind patterns, host plant availability, pest management practices, and limited gene flow between sites. Further evaluation of populations of this species is needed to determine if this shift is temporary and the impact on insecticide and Bt resistance management tactics.

## Figures and Tables

**Table 1:** Collection sites, Global Positioning System (GPS) coordinates, collection year and date, and the sample size of putative populations of *Helicoverpa zea* used in this study. The first two digits of the population name represents the collection year and the next two letters represent the collection site followed by the collection date. Rock Springs (RS), Landisville (LV).

Location	GPS Coordinates	Name of Putative Population	Collection Year	Collection Date	Sample Size
Rock Springs, PA	40°42′38.1″ N 77°57′52.2″ W	02RS-Aug12	2002	August 12	25
		02RS-Aug20	2002	August 20	24
		02RS-Aug27	2002	August 27	24
		02RS-Sep03	2002	September 03	117
		16RS-Sep01	2016	September 01	95
		18RS-Aug06	2018	August 06	33
		18RS-Aug23	2018	August 23	32
		18RS-Aug30	2018	August 30	30
Landisville, PA	40°07′04.7″ N 76°25′30.5″ W	05LV-Aug19	2005	August 19	39
		05LV-Aug24	2005	August 24	56
		05LV-Sep03	2005	September 3	95
		16LV-Sep02	2016	September 2	95
		18LV-Aug06	2018	August 6	24
		18LV-Aug13	2018	August 13	24
		18LV-Aug20	2018	August 20	23
		18LV-Aug27	2018	August 27	24
				Total	760

**Table 2:** Population specific inbreeding coefficient (*F*_IS_) indices (10,100 permutations) estimated for 16 putative populations of *Helicoverpa zea*. A negative value represents an essentially zero *F*_IS_ index. The first two digits of the population name represents the collection year and the next two letters represent the collection site followed by the collection date; Rock Springs (RS), Landisville (LV). Probability that a random *F*_IS_ will be greater than or equal to observed *F*_IS_ is given by *p*.

Population Number	Population Name	N	*F*_IS_	*p*
1	02RS-Aug12	23	0.1972	0.084
2	02RS-Aug20	24	0.2330	0.0551
3	02RS-Aug27	23	0.1734	0.1235
4	02RS-Sep03	114	−0.0350	0.6418
5	05LV-Aug19	35	−0.3623	0.9814
6	05LV-Aug24	52	* 0.1851	0.0270
7	05LV-Sep03	92	* 0.1467	0.0285
8	16LV-Sep02	83	0.0019	0.5021
9	16RS-Sep01	87	0.1325	0.0763
10	18LV-Aug06	23	0.0312	0.4629
11	18LV-Aug13	22	−0.0267	0.5750
12	18LV-Aug20	20	−0.0640	0.6333
13	18LV-Aug27	24	−0.0440	0.6034
14	18RS-Aug06	26	0.0247	0.4699
15	18RS-Aug23	29	0.1176	0.2498
16	18RS-Aug30	25	0.0988	0.2839

* Inbreeding coefficients that significantly deviated from zero at *p* ≤ 0.05.

**Table 3:** The results of analysis of molecular variance (AMOVA) of 16 *H. zea* collections grouped by various categories to calculate *F*_ST_ (between populations when there was only one group) or *F*_CT_ (among groups). Populations grouped together in each analysis are shown within square brackets.

Type of Grouping	Variance Component	% Total Variance	Φ-Statistic	*p*
1. All populations Group 1 [All populations	Among populations	4.89	*F*_ST_ = 0.0489	<0.000001
2. Grouped by collection Year Group 1 [02RS1, 02RS2] Group 2 [05LV1, 05LV2] Group 3 [16LV, 16RS3] Group 4 [18LV, 18RS]	Among groups Among populations within groups	4.84 7.29	*F*_CT_ =−0.04836 *F*_ST_ = 0.078669	=0.07871 <0.000001
3. Grouped by collection date Group 1 [02RS-Aug12, 18LV-Aug13 18RS-Aug06, 18LV-Aug06] Group 2 [02RS-Aug20, 18LV-Aug20 05LV-Aug19, 05LV-Aug24 18RS-Aug23] Group 3 [18RS-Aug30, 16RS-Sep01 02RS-Sep03, 16LV-Sep02, 05LV-Sep03] Group 4 [02RS-Aug27, 18LV-Aug27]	Among groups Among populations within groups	1.85 10.34	*F*_CT_ = 0.01847 *F*_ST_ = 0.10539	=0.11101 <0.000001
4. Grouped by the proportion of Cluster assignment of genotypes in the collection based on STRUCTURE results (Figure 3A,B)				
Group 1 [C1-02RS2, C1-18LV] Group 2 [C2-02R1, C2-02R2 05LV-Aug19, 05LV-Aug24 18RS-Aug23] Group 3 [C3-16LV, C3-16RS. C3-18RS]	Among groups Among populations within groups	0.45 8.21	IF_CT_ = 0.00583 *F*_ST_ = 0.10799	=0.41673 <0.000001
5. Grouped by genetic cluster assignment Group 1[Cluster1, Cluster 2, Cluster 3]	Among clusters	4.85	*F*_ST_ = 0.05283	<0.000001

## Supplementary Materials

The following are available online at https://www.mdpi.com/2075-4450/11/8/463/s1: Figure S1: The number of loci linked with 85 loci in putative *H. zea* populations collected in Landisville and Rock Springs, Pennsylvania. Figure S2: Result of the non-prior population DAPC analysis based on differences between successive values of Bayesian information criterion summary statistics and successive cluster assignment from repeated DAPC runs from K = 1 to 40. Figure S3. Major storms in 2018. The map downloaded from the National Weather Service website (https://www.ncdc.noaa.gov/monitoring-content/sotc/tropical-cyclones/2018/annual/tws_atl_latest.gif; accessed on 6 July 2020) indicates the dates of all named hurricanes in 2018 and color-coded paths of the named hurricanes. Parameter options selected to configure various plugins used for Genotype-by-Sequencing (GBS) analysis. Plugin name, option, value, and a brief description of the function of the plugin are listed on each column, Table S1; Single nucleotide polymorphism (SNP) loci developed for *H. zea* using the genome assembly, Table S2; Genotype data for all loci for all insects from putative populations obtained for *H. zea* population genetics study, Table S3; Genotype data for 85 loci for 702 *H. zea* used in population genetics study, Table S4; Mean and locus-by-locus observed and expected heterozygosities for putative populations, Tables 5a and 5b; Total number and mean of transitions, transversions, substitutions, private substitution sites, and the molecular diversity for 16 putative *H. zea* populations, Table S6; Locus by locus exacts tests for Hardy–Weinberg equilibrium, Table S7; Exact test (global) *p*-values for sample differentiation based on genotype frequencies, Table S8; Pairwise *F*_ST_ value estimates and significant(*p* ≤ 0.01) *F*_ST_ values for *H. zea* populations collected from Landisville and Rock Springs, PA, Table S9; Nei’s genetic distance (d) and the allelic differences between pairs of *H. zea* populations and the number of differences within each population, Table S10; The number of loci linked with 85 loci in putative *H. zea* populations, Table S11.
